# Prevalence and characteristics of transient myocardial thickening in cats with hypertrophic cardiomyopathy phenotypes

**DOI:** 10.1080/01652176.2025.2593357

**Published:** 2025-12-08

**Authors:** Sin-Wook Park, Keon Kim, Young-Jae Lee, Yoon-Jung Do, Woong-Bin Ro, Chang-Min Lee

**Affiliations:** a Department of Veterinary Internal Medicine, College of Veterinary Medicine and BK21 FOUR program, Chonnam National University, Gwangju, Korea; b Re-born Animal Medical Center, Busan, Korea; c Animal Diseases & Health Division, National Institute of Animal Science, Rural Development Administration, Wanju-gun, Korea

**Keywords:** Congestive heart failure, criteria, feline, heart diseases, myocardial diseases

## Abstract

This study aimed to propose new diagnostic criteria and provide detailed descriptions of cats diagnosed with transient myocardial thickening (TMT) using a retrospective design. HCM phenotype cases were defined as cats with echocardiographic findings of a maximum left ventricular wall thickness (LVWT) ≥6 mm on at least one presentation. TMT was defined as an increased LVWT ≥6 mm on at least two presentations, a subsequent decrease in LVWT of more than 25% within 6 months, no subsequent increase in left atrial-to-aortic root ratio (LA/Ao) ≥20% from baseline, and a fractional shortening ≤30%. A total of 145 cats had HCM phenotypes. Among cats that underwent serial echocardiography (*n* = 53), 16 were diagnosed with TMT and 34 with HCM. At presentation, the maximum LVWT was 8.3 ± 1.7 mm in cats with TMT, and decreased to 5.4 ± 0.9 mm after a median of 3.5 months [1–6 months]. Two cats initially classified as TMT were later identified as TMT on HCM based on persistent LV thickening, and all cats with TMT survived until the time of publication. The prevalence of TMT may be higher than expected when including asymptomatic TMT and TMT on HCM. Clinicians should be aware of the possibility of dynamic changes in myocardial thickness in cats.

## Introduction

The hypertrophic cardiomyopathy (HCM) phenotype is the most common form of feline heart disease, with a prevalence of approximately 15%. (Payne et al. 2015) This primary myocardial disorder is characterized by a hypertrophied non-dilated left ventricle in the absence of an obvious cause of left ventricular (LV) hypertrophy include hormonal diseases such as acromegaly, hyperthyroidism, systemic hypertension, hypovolemia, or transient myocardial thickening (TMT). (Trehiou‐Sechi et al. 2012; Luis Fuentes et al. 2020).

Among them, TMT is characterized by reversible myocardial thickening and has been reported in cats to cause an increased left atrial (LA) size and congestive heart failure (CHF). (Novo Matos et al. 2018; Romito et al. 2023; Wang and Seo 2024) Although the exact mechanism of development of TMT is not known, antecedent events may include spaying/neutering, surgery, burn injuries, or infections, while in some cases no clear trigger is identified. (Novo Matos et al. 2018; Romito et al. 2023; Wang and Seo 2024; Joung et al. 2024; Sharpe et al. 2020) In human medicine, myocarditis or stress-induced (Takotsubo) cardiomyopathy are associated with transient myocardial edema, which may represent the basis of reverse remodeling in feline TMT. (Romito et al. 2023; Gupta and Gupta 2018; Hiramitsu et al. 2001).

Recent studies have defined TMT by confirming an initially increased left ventricular wall thickness (LVWT; diastolic left ventricular wall thickness, LVWTd ≥6 mm) and a reduced LVWT (<5.5 mm) on follow-up echocardiography. (Novo Matos et al. 2018; Romito et al. 2023; Joung et al. 2024; Sharpe et al. 2020; Romito et al. 2022) In one illustrative case, a cat with primary HCM showed significant hypertrophy and CHF but later exhibited decreased LVWT (although still ≥6 mm) and reduced LA size on follow-up echocardiography; this cat remained stable after cardiac medications were discontinued. In addition, cases of Takotsubo cardiomyopathy or myocarditis on HCM have been reported in human medicine. (Frustaci et al. 2007; Abozenah et al. 2021) Therefore, new criteria for the diagnosis of TMT should include a transient form of myocardial thickening in cats with HCM.

To the best of our knowledge, there are no reports indicating the prevalence of TMT among cats with HCM phenotypes. Therefore, the purpose of this study was to determine the prevalence of TMT among cats with HCM phenotypes using revised diagnostic criteria and to describe clinical features, diagnostic findings, treatments, and outcomes. We also compared the clinical and echocardiographic characteristics of cats with TMT and those with HCM to identify distinguishing features.

## Materials and methods

### Animals

Medical records from Reborn Animal Referral Medical Center from April 2021 to March 2024 were collected, and cats were included in the study if they underwent echocardiography. Data collected included age, breed, sex, body weight, concurrent medical conditions, and results of physical examinations, clinical pathology, and diagnostic imaging. Hospitalization duration, survival to discharge, and treatments administered were also recorded. Follow-up information was obtained from medical records and by telephone interviews with owners.

### Animal groups

HCM phenotype cases were defined as cats with echocardiographic examinations with a maximum LVWTd ≥ 6 mm on at least one presentation. (Kittleson and Côté 2021) Among cats classified as having HCM phenotypes, those that underwent at least two echocardiographic examinations were evaluated for classification into the TMT and HCM groups. Cats diagnosed with concurrent diseases known to cause secondary myocardial thickening (e.g. hyperthyroidism, hypersomatotropism, systemic hypertension, dehydration, congenital heart diseases such as aortic stenosis) and/or the administration of drugs capable of inducing an increase in LVWT (e.g. corticosteroids) were excluded from both the TMT and HCM groups. (Romito et al. 2023; Campbell and Kittleson 2007)

TMT was defined as an increased LVWT (LVWTd ≥6 mm) at baseline, followed by *a* > 25% reduction in LVWT—either in the interventricular septum (IVS) or the left ventricular free wall (LVFW)—within six months of the first abnormal finding. Baseline was defined as the initial echocardiographic examination showing increased LVWT. Cats were excluded if they showed ≥20% increase in left atrium to the aorta (LA/Ao) compared with baseline, fractional shortening ≤30% (normal >30%), or focal LV wall thinning with hypokinetic or dyskinetic motion on the final examination. (Novo Matos et al. 2018; Chetboul et al. 2003; Payne et al. 2013) Within the TMT group, a subset of cats exhibited dynamic myocardial remodeling, with substantial wall thinning and preserved systolic function, but maintained an LVWT ≥6.0 mm on follow-up. Although fulfilling the echocardiographic criteria for HCM, these cats were classified as TMT on HCM based on the observed myocardial regression distinct from typical static hypertrophy. The HCM group was defined as cats exhibiting features consistent with primary HCM, characterized by a persistently increased maximum LVWT (LVWTd ≥6 mm) documented across serial echocardiographic examinations over a period exceeding six months, without evidence of regression.

Cats with signs of CHF were diagnosed based on pulmonary edema within 7 d after the first identification of increased LVWT. Cats without evidence of either CHF or arterial thromboembolism (ATE) at presentation or during the 7-d observation period were classified as asymptomatic.

### Echocardiographic data

All echocardiographic examinations were performed by two board-certified veterinary radiologists and reviewed by one board-certified veterinary internist to confirm inclusion and exclusion criteria. The value recorded for each measurement consisted of the average of three cardiac cycles. The IVS and LVFW thicknesses were measured from a two-dimensional (2D) right parasternal long-axis (RPLA) 4-chamber view and a right parasternal short-axis (RPSA) view at the papillary muscle level as the average of the thickest end-diastolic segment in each view (RPLA and RPSA views). (Novo Matos et al. 2018) The end-diastolic frame was defined as the first frame after mitral valve closure in the RPLA view and as the timepoint in the cardiac cycle of the greatest LV internal diameter in the RPSA view. (Novo Matos et al. 2018; Wess et al. 2010) The maximal average end-diastolic wall thickness from both the IVS and LVFW on these two views was recorded, and the highest value was defined as the maximum LVWT. (Novo Matos et al. 2018) The LV internal diameter in diastole (LVIDd) was measured in 2D from the RPLA and RPSA views at the level of the chordae tendineae in an end-diastole. The LA/Ao was measured in 2D from the RPSA basilar view just after aortic valve closure (end-ventricular systole). (Novo Matos et al. 2018; Romito et al. 2023) The LV fractional shortening (LVFS%) was measured in M-mode from the RPSA view at the level of the papillary muscle. The presence of systolic anterior motion of the mitral valve (SAM) was assessed on 2D and color Doppler from a RPLA 5-chamber view as the systolic motion of the tip of the anterior mitral valve leaflet toward the IVS producing turbulent flow in the LV outflow tract and mitral regurgitation. (Novo Matos et al. 2018) The presence of spontaneous echocardiographic contrast (SEC) or a thrombus was assessed from RPLA and RPSA views at the heart base. (Novo Matos et al. 2018; Payne et al. 2013)

### Statistical analysis

All statistical data were analyzed using commercial software (IBM SPSS Statistics, version 27, IBM Co., United States). Data were tested for normality with the Shapiro-Wilk test. Descriptive statistics are reported as the mean ± standard deviation (SD) for normally distributed continuous variables and as the median (interquartile range, IQR) for non-normally distributed continuous variables. For comparisons between two groups, the Chi-square test or Fisher’s exact test was used for categorical variables, and an independent samples t-test or the Mann-Whitney U-test was used for continuous variables. In addition, the Kruskal-Wallis test was used for comparisons among four groups, and multiple Mann–Whitney U tests with Bonferroni correction were performed as post hoc tests after which statistical significance was determined. Paired t-tests or Wilcoxon signed-rank tests were utilized to compare echocardiographic data between the time of the first presentation and later presentations, depending on the distribution of the variables. A significance level of *p* < 0.05 was used to determine statistical significance.

## Results

### Study population and clinical data

**Figure 1.**
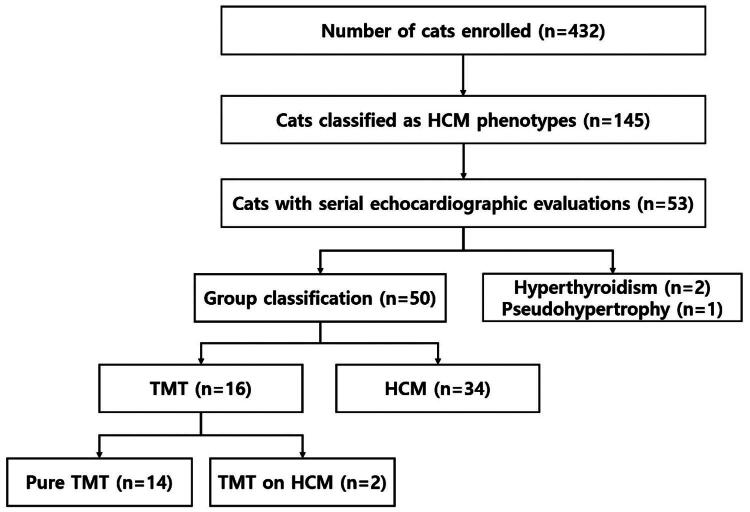
Flowchart illustrating the classification of cats enrolled in the study. A total of 432 cats underwent echocardiographic evaluation, of which 145 were classified as having HCM phenotypes. Among these, 53 cats underwent serial echocardiographic evaluations. Three cats were excluded from further classification due to the identification of hyperthyroidism (*n* = 2) or reversible pseudohypertrophy (*n* = 1). The remaining 50 cats were classified into the TMT and HCM groups based on serial echocardiographic findings: TMT group (*n* = 16) and HCM group (*n* = 34). Within the TMT group, 14 cats showed complete resolution of myocardial thickening (pure TMT), while 2 cats fulfilled the TMT criteria but maintained an LVWT ≥6.0 mm on follow-up and were designated as TMT on HCM. TMT, transient myocardial thickening; HCM, hypertrophic cardiomyopathy.

A total of 554 echocardiographic examinations from 432 cats were evaluated. Among them, 145 cats were classified as HCM phenotypes. Of the cats with HCM phenotypes, 53 underwent at least two echocardiographic examinations. After follow-up evaluations, 16 cats were classified as having TMT and 34 cats as having HCM (Figure 1). Among the 16 TMT cases, 14 cats exhibited complete resolution of myocardial thickening, consistent with pure TMT. Two cats met the echocardiographic criteria for TMT but maintained an LVWTd ≥6.0 mm at follow-up; these cases were designated as TMT on HCM and were included in the TMT group for analysis. Three cats that underwent serial evaluations could not be classified into either the TMT or HCM groups based on subsequent clinical assessments: two cats were diagnosed with hyperthyroidism, and one cat diagnosed with reversible pseudohypertrophy attributed to severe dehydration.

Domestic shorthair (DSH) cats were the most common breed across all groups. Among the 145 cats classified as having HCM phenotypes, 40 were DSH cats. Among the cats classified into the TMT and HCM groups after follow-up evaluations, 2 DSH cats were assigned to the TMT group and 9 DSH cats to the HCM group. The HCM phenotype population also included 18 Persians, 13 Scottish Folds, 9 British Shorthairs, 9 Turkish Angoras, 8 Russian Blues, 8 Siamese, 7 Ragdolls, 7 mixed breeds, 6 Munchkins, 5 Sphynx cats, 4 American Curls, 4 Norwegian Forest Cats, 3 Bengals, 2 American Shorthairs, and 2 Highlanders. The TMT population also included four British Shorthairs, three Scottish Folds, two American Curls, one Bengal, one Munchkin, one Norwegian Forest Cat, one Ragdoll, and on Turkish Angora. The HCM population also included five Turkish Angoras, four Persians, three Munchkins, two American Curls, two British Shorthairs, two Ragdolls, two Scottish Folds, one Bengal, one American Shorthair, one Russian Blue, one Siamese and one mixed breed.

The median age of cats with the HCM phenotype was 4.9 years [0.8–8.9 years]. Cats with TMT were younger (2.5 ± 1.8 years) than cats with HCM (7.1 [3.6–9.2] years), *p* < 0.001. There was no significant age difference between the sexes of cats with TMT. The mean body weight of cats with the HCM phenotype was 4.86 ± 1.43 kg. There was no significant difference in body weight between cats with TMT and HCM. A total of 102 were male (70.3%), and 99 of them were neutered; 43 cats were female (29.7%), and 42 of them were spayed. In the TMT group, 11 of 16 cats were male, and in the HCM group, 25 of 34 were male.

The characteristics of cats with TMT are summarized in Table 1. Antecedent events were identified in 62.5% of cats with TMT (10/16), and concurrent diseases were identified in 3 cats. The mean time to confirm an increased LVWT and/or the development of CHF after the antecedent events was 2.8 ± 1.0 d. The most frequent antecedent event was spaying/neutering (5 cats), followed by foreign body ingestion (2 cats). Other antecedent events included acute dyspnea, acetaminophen ingestion, and bathing. Concurrent diseases included feline infectious peritonitis (FIP) in two cats, and acute pancreatitis with hepatic lipidosis in one cat.

**Table 1.** Characteristics, concurrent diseases, antecedent events, number of echocardiographic examinations, follow-up duration, heart rate and echocardiographic parameters at presentation and follow-up in 16 cats with TMT.

Cat No.	Characteristics	Concurrent diseases and Antecedent event	CHF symptoms	Number of echocardiographic examinations and echocardiographic follow-up duration (months)	Heart rate^1^ (beats/min)	LVWT Max^1^ (mm)	IVSd MAX^1^ (mm)	LVFWd MAX^1^ (mm)	LVIDd^1^ (mm)	LVFS^1^ (%)	LA/Ao^1^	Heart rate^2^ (beats/min)	LVWT Max^2^ (mm)	IVSd MAX^2^ (mm)	LVFWd MAX^2^ (mm)	LVIDd^2^ (mm)	LVFS^2^ (%)	LA/Ao^2^
1	Munchkin, 7 m, SF, 2.4 kg	N	N	4/12	210	6.8	6.8	4.6	11.1	73	1.38	200	4.9	4.9	4.0	13.1	71	1.26
2	Scottish Fold, 3 y 7 m, SF, 5 kg	N	N	3/6	180	10.3	10.3	7.8	8.8	58	1.52	180	6.3	6.3	5.9	12.7	74	1.24
3	Ragdoll, 9 m, CM, 5.2 kg	FIP	N	3/7	132	6.8	6.8	4.4	9.9	69	1.24	150	4.3	4.3	4.1	16.2	55	1.32
4	Domestic Shorthair, 4 y 6 m, CM, 6.7 kg	Hepatic lipidosis, Acute pancreatitis	N	3/8	144	7.5	5.9	7.5	7.8	55	1.31	160	5.3	5.3	5.0	12.2	59	1.33
5	Norwegian Forest, 4 y 10 m, SF, 6.4 kg	Dyspnea	N	3/12	120	6.2	6.1	6.2	11.1	55	1.18	108	4.8	4.8	4.2	14.0	52	1.24
6	British Shorthair, 7 m, CM, 3.4 kg	FIP and Castration	CPE	3/6	132	7.2	7.2	7.1	12.5	49	1.48	160	4.5	4.5	4.0	14.0	45	1.15
7	American Curl, 10 m, SF, 2.7 kg	Ovariohysterectomy	CPE	3/12	108	6.7	6.7	6.6	7.5	42	2.33	120	4.9	4.9	4.8	9.5	69	1.47
8	British Shorthair, 10 m, CM, 3.4 kg	Foreign body ingestion	CPE	3/8	160	6.9	6.9	6.0	9.9	69	2.10	144	4.8	4.8	4.3	13.6	64	1.58
9	Turkish Angora, 2 y, SF, 4.6 kg	Foreign body ingestion	CPE	4/12	180	8.0	8.0	6.6	10.2	69	1.88	168	4.4	4.4	4.1	11.7	62	1.39
10	British Shorthair, 3 y 7 m, CM, 5.6 kg	N	CPE, PE	3/9	200	9.0	9.0	7.6	10.5	56	1.60	172	5.4	5.4	4.7	15.1	57	1.15
11	Domestic Shorthair, 2 y 9 m, CM, 6.4 kg	N	CPE, PE	5/24	150	10.3	9.0	10.3	9.0	69	1.98	180	5.8	5.8	5.3	13.9	62	1.67
12	British Shorthair, 6 y, CM, 5.5 kg	Acetaminophen ingestion	CPE, PE	4/24	200	11.4	9.5	11.4	8.0	58	1.79	180	7.0	7.0	6.8	12.6	46	1.15
13	American Curl, 4 y, CM, 6.6 kg	Castration	CPE, PE	5/18	144	9.2	7.4	9.2	11.5	43	1.99	160	5.4	4.9	5.4	14.1	52	1.47
14	Scottish Fold, 2 y 6 m, CM, 3.3 kg	Bathing	CPE	3/12	160	10.0	6.0	10.0	10.9	72	1.77	180	5.5	3.5	5.5	11.6	58	1.24
15	Bengal, 6 m, SF, 2.1 kg	Ovariohysterectomy	CPE, PE	8/33	180	6.7	6.3	6.7	8.0	53	1.68	160	4.3	4.0	4.3	12.7	46	1.56
16	Scottish Fold, 1 y 5 m, CM, 4.5 kg	Castration	CPE	4/12	220	9.5	9.5	7.7	12.6	57	2.09	200	5.6	5.6	4.8	13.8	60	1.29

CM, castrated male; CPE, cardiogenic pulmonary edema; FIP, feline infectious peritonitis; IVSd Max, maximal interventricular septal thickness in end-diastole; LAD, left atrial diameter; LA/Ao, left atrium to aortic root ratio; LVFS, left ventricular fractional shortening; LVIDd, left ventricular internal diameter in end-diastole; LVFWd Max, maximal left ventricular free wall thickness in end-diastole; LVWT Max, maximal left ventricular wall thickness; PE, pleural effusion; SF, spayed female; y, year; m, month.

1, initial echocardiogram; 2, final echocardiogram.

All cats survived without reoccurrence and discontinuation of cardiac medications, with a total follow-up duration (including telephone follow-up) of at least 15 months.

### Echocardiographic data

Cats with TMT and HCM had a median of 3 [3–4] echocardiographic examinations during the study period. The median maximum LVWT was 7.6 mm [6.7–8.6 mm], and the median LA/Ao was 1.88 [1.37–2.2] in cats with the HCM phenotype at the first presentation. A total of 70 cats with the HCM phenotype presented with either CHF, ATE, or both at the time of initial evaluation, and exhibited a higher LA/Ao (2.11 [1.9–2.4]) compared to asymptomatic cats (*n* = 75; 1.43 [1.22–1.78]; *p* < 0.001).

**Figure 2.**
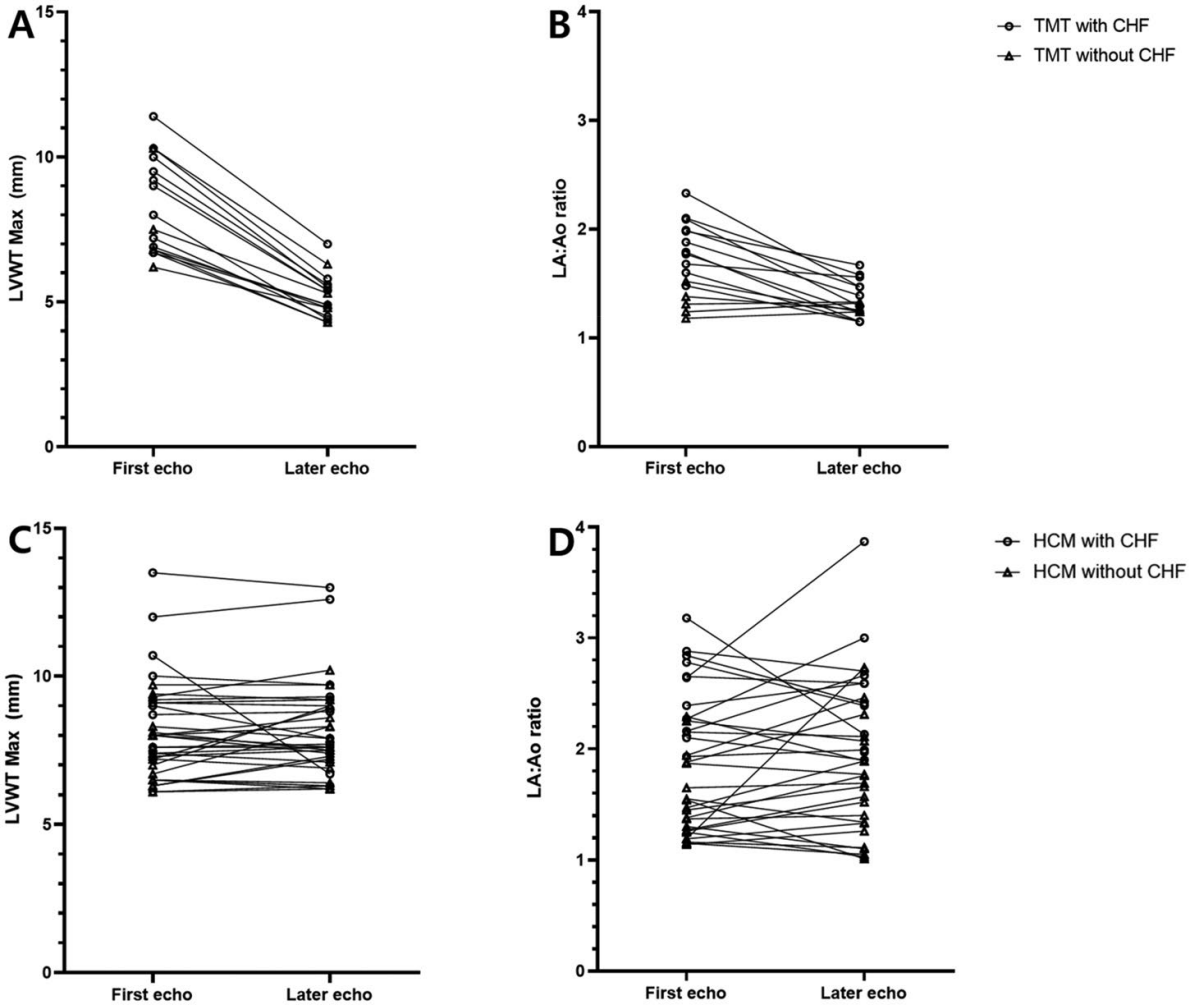
Maximum left ventricular wall thickness (LVWT max) (A, C) and left atrial to aortic root ratio (LA/Ao) (B, D) in cats with transient myocardial thickening (TMT) (A, B) and hypertrophic cardiomyopathy (HCM) (C, D) at presentation and on the final echocardiogram within one year after the first echocardiogram. In contrast to cats with HCM, significant decreases in the LVWT and LA/Ao over time were found in cats with TMT. Echo, echocardiogram; TMT, transient myocardial thickening; HCM, hypertrophic cardiomyopathy; LA/Ao, left atrial to aortic root ratio; LVWT max, maximum left ventricular wall thickness.

**Figure 3.**
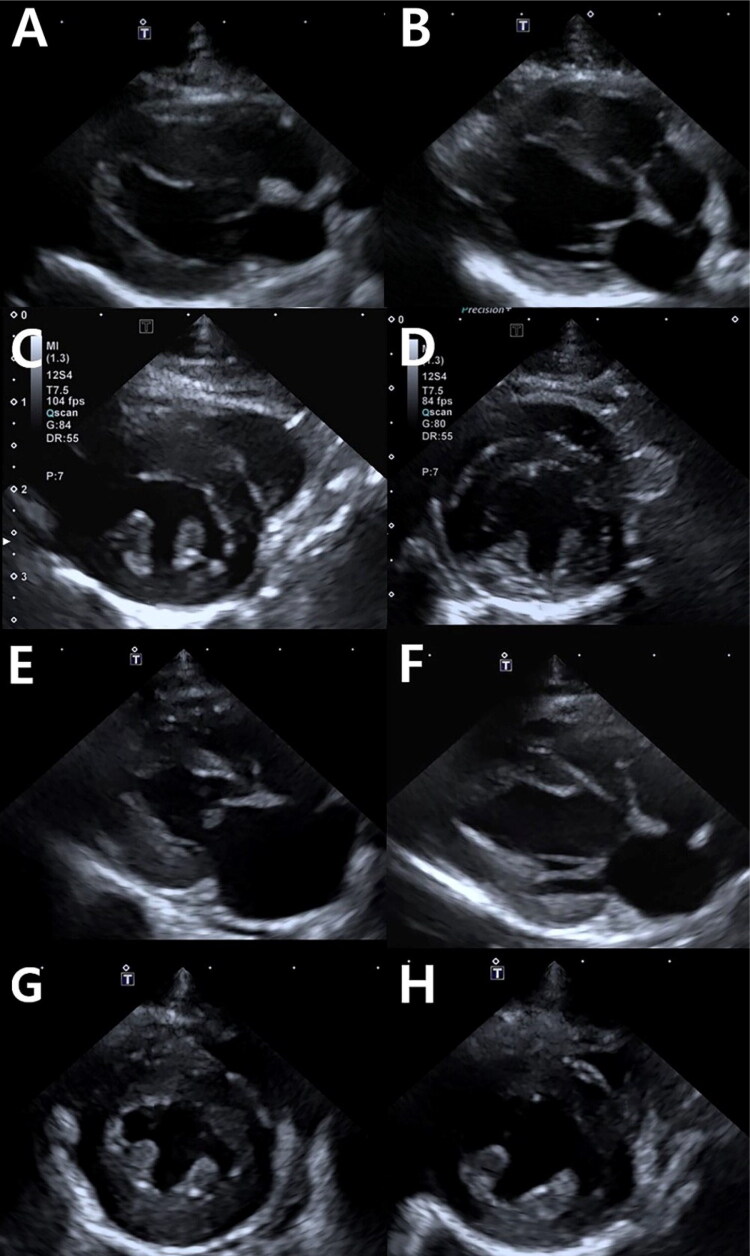
Echocardiography obtained in two cats from this study at the time of first presentation (A, C, E G) and one month later (B, D, F, H). One cat remained asymptomatic (A, B, C, D), and another cat (E, F, G, H) suffered from congestive heart failure (CHF). Right parasternal long-axis (A, B, E, F) and short-axis basilar views (C, D, G, H) in an end-diastolic frame. The initial increased left ventricular wall thickness (A, C, E, G) resolved, and the left atrial diameter was reduced in a cat suffering from CHF (E, F).

At presentation, the maximum LVWT was 8.3 ± 1.7 mm in cats with TMT. No significant differences were observed in IVS and LVFW thickness between the TMT and HCM groups. Similarly, at presentation, there was no significant difference in the LA/Ao between the TMT group and HCM group. In cats with TMT, after a median period of 3.5 months [1–6 months], a decrease in the LVWT was observed (Figures 2 and 3). The median decreases in the IVS and LVFW in the TMT group were 36% [28–39%] and 37% [28–41%] respectively. The median rates of change in the IVS and LVFW in the TMT group were 6% [2–12%] and 6% [3–13%] respectively. Likewise, the LA/Ao decreased over time in the TMT group (24% [8–29%]), while it showed no considerable change in the HCM group (Figure 2). In addition, the LVIDd significantly increased over time in the TMT group (*p* < 0.001).

In the TMT and HCM groups, the LVFW was more thickened and the LA/Ao was higher in cats with CHF than in asymptomatic cats on the initial echocardiogram (*p* < 0.05) (Table 2). In the TMT group, the final echocardiogram within one year after the first echocardiogram showed there were no significant differences in the IVS, LWWT, and LA/Ao between cats with CHF and cats without CHF (*p* > 0.05). There were no significant differences in the IVS, LVWT, LVFS, and LA/Ao between asymptomatic cats in the TMT group and HCM group at presentation. However, in cats with CHF, the LA/Ao was higher in the HCM group than TMT group (*p* < 0.001). There were no significant differences in the LVFS among groups.

**Table 2.** Clinical characteristics and echocardiographic variables in the TMT group and HCM group.

	TMT without CHF (*n* = 5)	HCM without CHF (*n* = 22)	TMT with CHF (*n* = 11)	HCM with CHF (*n* = 12)	P
Age (month)^1^	43 (9–54)^a^	87 ± 56^b^	27 ± 21^a^	87 ± 38^b^	<0.001**
Body weight	5.2 (5.1–6.4)	4.7 (4.1–6.4)	4.4 ± 1.5	4.4 ± 1.4	0.607
IVS^1^ (mm)	6.8 (6.1–6.8)	6.8 (6.3–7.6)	7.8 ± 1.3	7.3 ± 2.8	0.508
IVS^2^ (mm)	4.9 (4.8–5.3)^a^	7.0 ± 0.9^b^	5.0 ± 1.0^a^	7.6 ± 2^b^	<0.001**
LVFW^1^ (mm)	6.2 (4.6–7.5)^a^	6.4 ± 1.6^a^	8.1 ± 1.8^b^	8.8 ± 2.4^b^	0.029*
LVFW^2^ (mm)	4.2 (4.1–5)^a^	6.9 ± 1.8^b^	5.0 ± 0.8^a^	8.6 ± 2.3^c^	<0.001**
LVFS^1^	58 (54–69)	57 ± 12	58 ± 11	49 ± 10	0.059
LVFS^2^	59 (55–71)	53 ± 11	58 ± 8	46 ± 12	0.051
LA/Ao^1^	1.31 (1.24–1.38)^a^	1.42 (1.25–1.82)^a^	1.88 ± 0.24^b^	2.54 ± 0.37^c^	<0.001**
LA/Ao^2^	1.26 (1.24–1.32)^a^	1.53 ± 0.37^a^	1.37 ± 0.19^a^	2.51 ± 0.54^b^	<0.001**
SEC^1^ (yes)	0	0	0	3	
Pericardial effusion^1^	0	0	1	4	

1: initial echocardiogram, 2: final echocardiogram within one year after the first echocardiogram.

Descriptive statistics are reported as the mean ± standard deviation (SD) for normally distributed continuous variables and as the median (interquartile range, IQR) for non-normally distributed continuous variables.

* *p* < 0.05 and **p < 0.001 indicate a statistically significant difference.

^a,b,c^ Means within a row with different superscripts differ significantly (*p* < 0.05).

CHF, congestive heart failure; HCM, hypertrophic cardiomyopathy; IVS, interventricular septum; LA/Ao, left atrial to aortic root ratio; LVFS, left ventricular fractional shortening; LVFW, left ventricular free wall; SEC, spontaneous echocardiographic contrast; TMT, transient myocardial thickening.

The TMT and HCM groups were divided into two groups according to the presence or absence of CHF.

### Final diagnosis, treatment data, and outcome data

At presentation, a few asymptomatic cats with HCM were receiving medications that did not include diuretics. These treatments, which included clopidogrel (*n* = 5, 18.5%) and/or pimobendan (*n* = 5, 18.5%), were maintained throughout the study period. All cats with CHF, whether classified as HCM or TMT, were managed according to standard clinical practice with medications for CHF and prevention of ATE. These treatments included furosemide and clopidogrel for all cats (23/23 [100%]), pimobendan for 12/23 (52.2%), benazepril for 7/23 (30.4%), spironolactone for 5/23 (21.7%), and atenolol for 1/23 (4.3%). In all cats with HCM and CHF (12/12), cardiac medications were continued throughout the study or until death, and six died from recurrent CHF or ATE. In contrast, all cats with TMT and CHF (11/11) discontinued cardiac medications after resolution of CHF, and none experienced recurrence. At the time of manuscript preparation, all were alive with follow-up periods of at least 15 months. Two cats fulfilled the criteria for TMT on HCM and were analyzed within the TMT group.

## Discussion

This retrospective study evaluated 432 cats with echocardiographic evidence of HCM phenotypes, of which 145 were classified for further analysis. Among these, 53 cats underwent at least two echocardiographic examinations, allowing assessment of whether myocardial thickening persisted or resolved over time. After follow-up evaluations, 16 cats were classified as TMT and 34 as HCM, indicating a prevalence of approximately 30% among cats with repeated examinations and 11% among the overall HCM phenotype population. Because confirmation of TMT required serial echocardiography, the true prevalence may have been underestimated. For example, two cats developed CHF with increased LVWT after neutering and died during hospitalization before follow-up imaging, suggesting that additional TMT cases may not have been captured.

To minimize confounding factors, cats with concurrent endocrine disorders such as hyperthyroidism, concurrent cardiac abnormalities such as aortic stenosis, or administration of medications known to influence myocardial thickness (e.g. corticosteroids) were excluded from group classification. Secondary causes of apparent myocardial thickening, such as dehydration or systemic conditions including systemic hypertension, were also considered during case selection to avoid misclassification. These criteria were applied to ensure that only primary myocardial disease was included. In contrast, recent surgical procedures and potential infectious diseases were not used as exclusion criteria, since anesthesia, surgery, and infection may act as important triggers or underlying causes of TMT.

In previous feline studies, TMT has often been defined as an initially increased LVWT (≥6.0 mm) that subsequently decreased to <5.5 mm on follow-up echocardiography. (Novo Matos et al. 2018; Romito et al. 2023; Wang and Seo 2024; Joung et al. 2024Romito et al., 2022) In contrast, the present study defined TMT by a relative reduction in wall thickness of >25%, a threshold selected to account for known measurement variability in echocardiographic assessments. (Chetboul et al. 2003) Using this definition, two cats were diagnosed as TMT despite maintaining an LVWT ≥6.0 mm at follow-up. Both showed a 39–40% reduction in LVWT together with myocardial wall thinning, normalization of LA/Ao, and preserved systolic function, and remained clinically stable without recurrence of CHF after discontinuation of cardiac medications. These cases would not have fulfilled the conventional criterion of a reduction to <5.5 mm, highlighting the need for a broader definition of TMT. Importantly, such cases illustrate that transient myocardial thickening can occur even in cats with pre-existing HCM, a phenomenon also recognized in human medicine with overlap between primary cardiomyopathy and stress-induced or inflammatory cardiomyopathies. (Frustaci et al. 2007; Abozenah et al. 2021) Our findings therefore suggest that the diagnostic concept of TMT in cats should be expanded to include these transient forms occurring in association with HCM.

Both TMT and HCM groups showed increased LV wall thickness at the initial echocardiographic assessment, often accompanied by LA enlargement and clinical signs of CHF. However, their subsequent clinical courses differed substantially. Cats with TMT exhibited a reduction in LVWT with concurrent improvement in LA/Ao and preserved systolic function, whereas cats with HCM showed persistently increased LVWT, progressive LA enlargement, and, in some cases, reduced systolic function. From a clinical perspective, this distinction is important: cats with previously asymptomatic HCM that develop CHF are often presumed to have progressive, irreversible disease requiring lifelong cardiac therapy, yet our findings indicate that in some cases CHF may instead be attributable to transient myocardial thickening. Recognizing this scenario is clinically relevant, as it allows discontinuation of cardiac medications once myocardial thickening and atrial size normalize, thereby avoiding unnecessary long-term treatment.

In previous reports, most cats with TMT presented with CHF, and limited information was available for asymptomatic cases. (Romito et al. 2023) In contrast, 5 of 16 cats with TMT in the present study were initially asymptomatic. One illustrative case (Cat No. 9) initially showed myocardial thickening without clinical signs but developed CHF after surgery. Serial echocardiography later confirmed that this case fulfilled the criteria for TMT. This observation suggests that subclinical TMT, while not requiring immediate treatment, may progress to overt CHF following anesthesia or surgical stress. Recognition of such cases may be clinically important, particularly in young cats with increased LVWT and recent antecedent events, underscoring the need for perioperative caution.

Infectious diseases may also contribute to TMT. Myocarditis associated with Bartonella, Toxoplasma, feline immunodeficiency virus, and FIP have been reported in cats. (Romito et al. 2022; Ernandes et al. 2019; Rolim et al. 2016; Joseph et al. 2018) One previous study described a cat with FIP that developed CHF with increased LVWT after anesthesia, although follow-up echocardiography was not performed because the cat was euthanized. (Ernandes et al. 2019) In the present study, two cats with TMT were diagnosed with FIP; one remained asymptomatic, and the other developed CHF following castration. Although histopathology was not available, these cases raise the possibility that FIP-associated myocarditis may have been responsible for the transient myocardial changes. While other infectious etiologies such as bartonellosis were not systematically excluded, all cats in this study were strictly indoor, making such infections less likely. This possibility underscores the importance of comprehensive diagnostic screening for infectious agents known to affect the cardiovascular system in future studies.

In this study, the LA/Ao was the only echocardiographic parameter that could distinguish between the TMT and HCM groups with CHF on initial echocardiography, and the LA/Ao decreased over time in cats with TMT. These results indicate an acute myocardial injury that occurred before the left atrium had time to remodel to adjust for the increased atrial filling pressures, which may lead to a rapid increase in LA pressure and pulmonary capillary pressure causing acute pulmonary edema. (Novo Matos et al. 2018; Oyama et al. 2004) Cats with a higher LA/Ao are more likely to have HCM, but there was significant overlap in the LA/Ao between the two groups; therefore, this feature alone may not reliably differentiate cats with TMT from those with HCM. (Zamorska and Grushanska 2022)

In one cat classified as HCM, LVWT decreased by more than 25% during follow-up, accompanied by reduced systolic function and LA enlargement, a pattern consistent with end-stage HCM rather than TMT. (White 2015) This finding highlights the importance of evaluating LV function and LA/Ao alongside wall thickness when diagnosing TMT, as myocardial thinning in end-stage HCM can mimic regression. The median time from presentation to confirmed diagnosis of TMT was 3.5 months, comparable to previous reports of 1.5–3.3 months. (Novo Matos et al. 2018; Romito et al. 2023) Most cases showed decreased myocardial thickness by the second echocardiographic examination, emphasizing the value of serial imaging at relatively short intervals. Similar to prior studies, cats with TMT were younger (mean age 2.4 years) than those with HCM (median age 7.1 years), suggesting that age may play a role in disease expression. (Novo Matos et al. 2018; Romito et al. 2023)

Although heart rate in TMT cats did not change significantly between the initial and follow-up examinations, several cats showed a decrease that may have partially influenced the reduction in wall thickness. Experimental studies in healthy cats have shown that tachycardia can transiently increase apparent myocardial thickness through shortened diastolic filling time. (Sugimoto et al. 2017) Moreover, stress-related sympathetic activation and catecholamine release can elevate heart rate and enhance myocardial contractility, contributing to both true and pseudo-hypertrophy. (Novo Matos et al. 2018; Romito et al. 2023; Sugimoto et al. 2017) In this study, however, the magnitude of wall-thickness regression often exceeded what could be explained by heart rate variation alone, suggesting genuine myocardial remodeling. Although current diagnostic criteria for HCM do not consider heart rate, similar rate-dependent variation may also warrant attention in the evaluation of TMT.

There are several limitations to this study. First, as a retrospective investigation, diverse echocardiographic parameters (e.g. tissue Doppler imaging, speckle-tracking echocardiography) were not evaluated systematically. Second, cardiac and inflammatory biomarkers, electrocardiography, and histopathology were not consistently available, precluding systematic screening for infectious etiologies such as FIP-associated myocarditis. Third, echocardiography was performed at different times after the onset of CHF, and few cats in critical condition were treated with diuretics prior to the initial echocardiographic evaluation. In addition, some cats with HCM received pimobendan, which could have influenced cardiac contractility. However, because the primary comparisons focused on structural parameters such as LV wall thickness and LA size, the potential impact on the main study outcomes is considered limited. Moreover, although we ruled out dehydrated cats, the inclusion of some cats with clinically inapparent dehydration potentially associated with some degree of pseudohypertrophy cannot be completely excluded. Finally, intraobserver and interobserver measurement repeatability were not formally assessed in this study. However, standardized imaging protocols, averaging of measurements across three cardiac cycles, and a strict definition requiring >25% reduction in LVWT for classification were applied to minimize the potential impact of measurement variability. Nonetheless, this lack of quantitative assessment represents a limitation and may have influenced the classification of TMT, particularly in asymptomatic cats.

## Conclusions

TMT can mimic HCM at the initial examination and may also occur in cats with pre-existing HCM, potentially worsening their condition. Recognizing TMT as a distinct and sometimes reversible condition is important for clinical decision-making. Clinicians should be aware of the dynamic nature of myocardial thickness in cats, and further studies are warranted to establish standardized diagnostic criteria.

## References

[CIT0001] Abozenah M et al. 2021. Concurring hypertrophic cardiomyopathy and Takotsubo cardiomyopathy: assessment and management. Heart Lung. 50(4):546–557. 10.1016/j.hrtlng.2020.10.006 33143911

[CIT0002] Campbell FE, Kittleson MD. 2007. The effect of hydration status on the echocardiographic measurements of normal cats. Vet Intern Med. 21(5):1008–1015. 10.1111/j.1939-1676.2007.tb03057.x 17939557

[CIT0003] Chetboul V et al. 2003. Effects of inter‐and intra‐observer variability on echocardiographic measurements in awake cats. J Vet Med A Physiol Pathol Clin Med. 50(6):326–331. 10.1046/j.1439-0442.2003.00546.x 12887627

[CIT0004] Ernandes MA et al. 2019. Feline coronavirus-associated myocarditis in a domestic longhair cat. JFMS Open Rep. 5(2):2055116919879256. 10.1177/2055116919879256 31636915 PMC6787879

[CIT0005] Frustaci A et al. 2007. Myocarditis in hypertrophic cardiomyopathy patients presenting acute clinical deterioration. Eur Heart J. 28(6):733–740. 10.1093/eurheartj/ehl525 17309901

[CIT0006] Gupta S, Gupta MM. 2018. Takotsubo syndrome. Indian Heart J. 70(1):165–174. 10.1016/j.ihj.2017.09.005 29455773 PMC5902911

[CIT0007] Hiramitsu S et al. 2001. Transient ventricular wall thickening in acute myocarditis a serial echocardiographic and histopathologic study. Jpn Circ J. 65(10):863–866. 10.1253/jcj.65.863 11665789

[CIT0008] Joseph J, Oxford E, Santilli R. 2018. Transient myocardial thickening in a Bartonella henselae–positive cat. J Vet Cardiol. 20(3):198–203. 10.1016/j.jvc.2018.04.003 29730195

[CIT0009] Joung Y, Ahn H, Choi J, Yun Y, Song W-J. 2024. Transient Myocardial Thickening in a 4-year-old Korean Domestic Shorthair Cat. J Vet Clin. 41(2):106–111. 10.17555/jvc.2024.41.2.106

[CIT0010] Kittleson MD, Côté E. 2021. The feline cardiomyopathies: 2. Hypertrophic cardiomyopathy. J Feline Med Surg. 23(11):1028–1051. 10.1177/1098612X211020162 34693811 PMC8642168

[CIT0011] Luis Fuentes V et al. 2020. ACVIM consensus statement guidelines for the classification, diagnosis, and management of cardiomyopathies in cats. J Vet Intern Med. 34(3):1062–1077. 10.1111/jvim.15745 32243654 PMC7255676

[CIT0012] Novo Matos J et al. 2018. Transient myocardial thickening in cats associated with heart failure. J Vet Intern Med. 32(1):48–56. 10.1111/jvim.14897 29243322 PMC5787177

[CIT0013] Oyama MA, Sisson DD, Bulmer BJ, Constable PD. 2004. Echocardiographic estimation of mean left atrial pressure in a canine model of acute mitral valve insufficiency. Vet Int Med. 18(5):667–672. 10.1111/j.1939-1676.2004.tb02604.x 15515583

[CIT0014] Payne JR et al. 2013. Prognostic indicators in cats with hypertrophic cardiomyopathy. J Vet Intern Med. 27(6):1427–1436. 10.1111/jvim.12215 24134821

[CIT0015] Payne JR, Brodbelt DC, Fuentes VL. 2015. Cardiomyopathy prevalence in 780 apparently healthy cats in rehoming centres (the CatScan study). J Vet Cardiol. 17 Suppl 1: s 244–S257. 10.1016/j.jvc.2015.03.008 26776583

[CIT0016] Rolim VM, Casagrande RA, Wouters ATB, Driemeier D, Pavarini SP. 2016. Myocarditis caused by feline immunodeficiency virus in five cats with hypertrophic cardiomyopathy. J Comp Pathol. 154(1):3–8. 10.1016/j.jcpa.2015.10.180 26797583 PMC7094316

[CIT0017] Romito G et al. 2023. Transient myocardial thickening: a retrospective analysis on etiological, clinical, laboratory, therapeutic, and outcome findings in 27 cats. J Vet Cardiol. 50:51–62. 10.1016/j.jvc.2023.09.001 37924558

[CIT0018] Romito G, Fracassi F, Cipone M. 2022. Transient myocardial thickening associated with acute myocardial injury and congestive heart failure in two Toxoplasma gondii-positive cats. JFMS Open Rep. 8(2):20551169221131266. 10.1177/20551169221131266 36339325 PMC9629561

[CIT0019] Sharpe AN, Gunther-Harrington CT, Epstein SE, Li RH, Stern JA. 2020. Cats with thermal burn injuries from California wildfires show echocardiographic evidence of myocardial thickening and intracardiac thrombi. Sci Rep. 10(1):2648. 10.1038/s41598-020-59497-z 32060317 PMC7021798

[CIT0020] Sugimoto K, Fujii Y, Ogura Y, Sunahara H, Aoki T. 2017. Influence of alterations in heart rate on left ventricular echocardiographic measurements in healthy cats. J Feline Med Surg. 19(8):841–845. 10.1177/1098612X16661374 27502088 PMC11104112

[CIT0021] Trehiou‐Sechi E et al. 2012. Comparative echocardiographic and clinical features of hypertrophic cardiomyopathy in 5 breeds of cats: a retrospective analysis of 344 cases (2001–2011). Vet Intern Med. 26(3):532–541. 10.1111/j.1939-1676.2012.00906.x 22443341

[CIT0022] Wang Y, Seo J. 2024. Transient myocardial thickening after routine ovariohysterectomy in a 15‐month‐old Ragdoll cat. J Small Anim Pract. 65(8):648–652. 10.1111/jsap.13722 38444263

[CIT0023] Wess G, Sarkar R, Hartmann K. 2010. Assessment of left ventricular systolic function by strain imaging echocardiography in various stages of feline hypertrophic cardiomyopathy. J Vet Intern Med. 24(6):1375–1382. 10.1111/j.1939-1676.2010.0586.x 20738767

[CIT0024] White AJ. 2015. End-stage hypertrophic cardiomyopathy in a cat. Can Vet J. 56(5):509. 25969586 PMC4399740

[CIT0025] Zamorska T, Grushanska N. 2022. Cardiogenic and non-cardiogenic pulmonary oedema in a domestic cat: pathological mechanisms, differential diagnosis, and treatment. Ukr J Vet Sci. 13(1):34–43. 10.31548/ujvs.13(1).2022.34-43

